# Is FSH combined with equine chorionic gonadotropin able to modify lipid metabolism in bovine superstimulated antral follicles?

**DOI:** 10.1590/1984-3143-AR2023-0063

**Published:** 2024-07-05

**Authors:** Priscila Helena Santos, Fernanda Fagali Franchi, Sarah Gomes Nunes, Patricia Kubo Fontes, Alan Brunholi Giroto, Fernanda Mani, Anthony César de Souza Castilho

**Affiliations:** 1 Departamento de Biofísica e Farmacologia, Instituto de Biociências, Universidade Estadual Paulista “Júlio de Mesquita Filho”, Botucatu, SP, Brasil; 2 Departamento de Ciência Animal, Universidade do Oeste Paulista, Presidente Prudente, SP, Brasil; 3 Departamento de Química e Bioquímica, Instituto de Biociências, Universidade Estadual Paulista “Júlio de Mesquita Filho”, Botucatu, SP, Brasil

**Keywords:** triglycerides, follicle microenvironment, superovulation, gene expression, bovine

## Abstract

Lipid metabolism is essential for ensuring oocyte maturation and embryo development. β-Oxidized fatty acids (FA) are a potent source of energy for cells, particularly for bovine somatic follicular cells. Superstimulatory protocols using follicle stimulating hormone (FSH) or FSH combined with equine chorionic gonadotropin (eCG) are capable of stimulating the follicular microenvironment and drive the expression of biomarker genes associated with lipid metabolism in the cumulus-oocyte complex (COC) for better embryo development. In this study, we assesed the effects of FSH and FSH/eCG protocols on the expression of genes related to lipid metabolism in bovine granulosa cells (GCs). Further, we measured triglyceride levels in follicular fluid (FF) obtained from both superstimulatd and non-superstimulated cows (synchronized cows). In summary, superstimulation with gonadotropins maintained the TG levels in bovine FF and ensured GCs mRNA abundance of *ACSL1, ACSL3, ACSL6, SCD, ELOVL5, ELOVL6, FASN, FADS2,* and *SREBP1.* We, however, found the abundance of *CPTIB* mRNA to be lower in GCs obtained from cows subjected to FSH/eCG protocols than synchronized cows. In conclusion, the findings of this study showed that ovarian superstimulation around the preovulatory phase has a mild impact on the lipid metabolism in GCs.

## Introduction

Follicle development involves a range of coordinated processes to reach an oocyte able to be fertilized and become an embryo (Adams et al., 1992; Fortune, 1994; Drummond, 2006; Aller et al., 2013). After follicle deviation, the dominant follicle cells continue to proliferate and differentiate into granulosa cells (GCs) under the influence of luteinizing hormone (LH) and estradiol. In this context, the cells express a variety of genes depending on the specific estral phase, and the follicular environment provides to oocyte the required substrates, such as growth factors and constituent molecules to reach the ovulation (Bao and Garverick, 1998; Webb et al., 1999; Orisaka et al., 2006; Aller et al., 2013).

Among the many metabolic pathways involved in follicular development, lipid metabolism is fundamental to oocyte quality (Collado-Fernandez et al., 2012; Dunning et al., 2010; Elis et al., 2015; Warzych et al., 2017). Fatty acids (FAs) are a class of lipids involved in the prostaglandin synthesis and structural components of plasmatic membrane (Carvalho and Caramujo, 2018). In addition, FA β-oxidation provides a potent source of energy that enables cell development and proliferation and FA β-oxidation, such as that of palmitate, can generate 106 ATP molecules, whereas glucose oxidation generates only 30 ATP molecules (Berg et al., 2002; Fu et al., 2021).

Follicular lipid content and lipid profile is dependent on the follicular growth phase and follicular compartment (theca and GCs, follicular fluid, and oocyte) (Collado-Fernandez et al., 2012; Annes et al., 2019; Bertevello et al., 2018) and, it is related to the cellular function (15). Cells store lipids as droplets; it serves as an energy reserve, with triglycerides (TG) being the main component (Aardema et al., 2011; Cran, 1985; Sturmey et al., 2009). Alteration of the lipid profile in the follicular microenvironment could impact cellular function, oocyte quality and embryo development.

Superstimulatory protocols using follicle stimulating hormone (FSH) or FSH combined with equine chorionic gonadotropin **(**eCG), are known to modulate intracellular LH receptor (LHR) signaling in GC pathways in preovulatory follicles (Castilho et al., 2014) as well as mediate steroidogenic capacity in GCs (Santos et al., 2018) and lipid profile in follicular fluid (FF) (Santos et al., 2017). Furthermore, Franchi et al. (2019) demonstrated that the FSH/eCG these protocols may also affect lipid metabolism in cumulus-oocyte complex (COC) and embryos. Considering the importance of lipid metabolism in reproduction, mainly in acquiring quality oocyte, we assessed the effect of protocols on the expression of genes in the lipid metabolism pathways in GCs of preovulatory follicles, either subjected to ovary superstimulation or not; further, we also assessed the effect of these protocols on TG levels in bovine FF.

## Methods

### Experimental design

**Figure 1:**
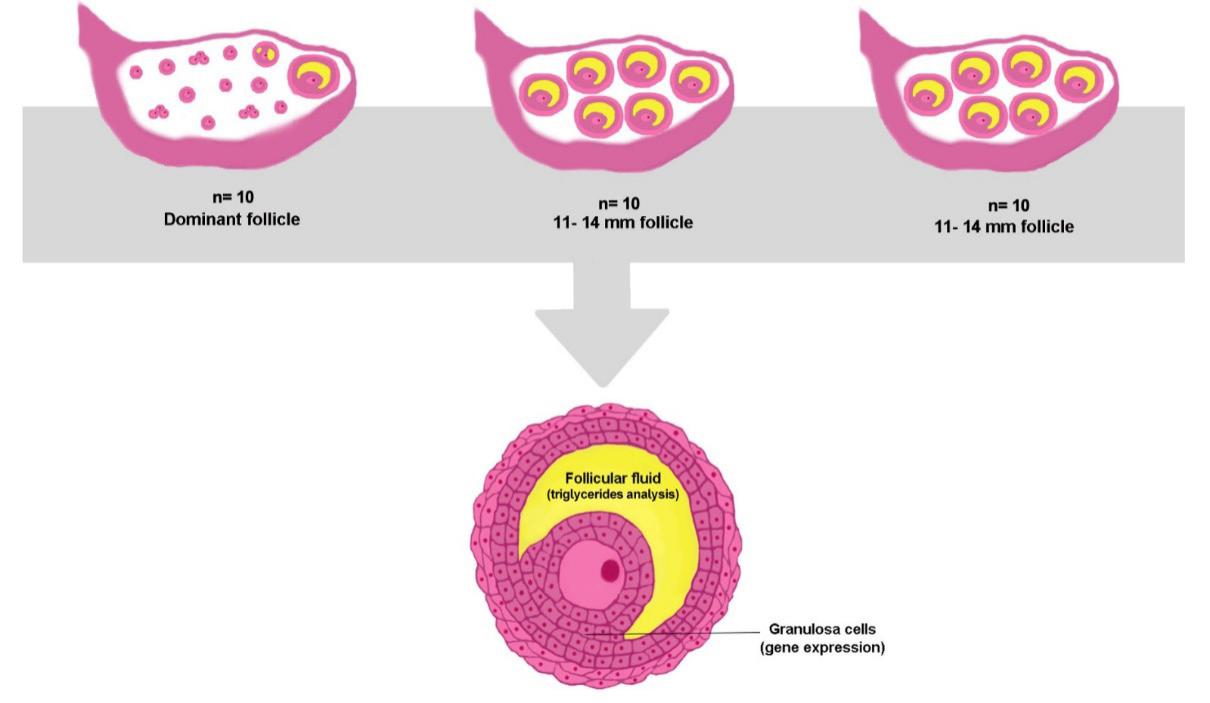
Experimental design to investigate the effects of FSH and FSH/eCG in Nelore cows on TG levels from FF, and the relative abundance of target genes in GCs from cows subjected to superstimulatory protocols or synchronized group (n=10/group).

To gain insight into the effects on lipid metabolism in GCs from preovulatory follicles subjected to ovarian superstimulation, an experiment was designed (Figure 1). All experimental animals were treated according to the Brazilian animal protection laws. This study was conducted on a farm located in Santa Cruz do Rio Pardo (São Paulo, Brazil; latitude 22º 53’ 56″; longitude 49º 37’ 57″; altitude 467 m). The cows were maintained on pastures (*Brachiaria brizantha*) with *ad libitum* access to water and mineral supplements.

Samples used in this study, the animal conditions, and superstimulatory protocols have been described previously (Castilho et al., 2014; Santos et al., 2017; Santos et al., 2018; Franchi et al., 2019; Fontes et al., 2014). The local *Ethics Committee on Animal Use* from the Institute of Biosciences (University of São Paulo State (UNESP), Botucatu, São Paulo, Brazil) approved the experiments (protocol number: 379). Animals were separated into three groups: ovarian superstimulatory protocols, FSH or FSH/eCG protocol, and synchronized cows (control).

### Ovarian superstimulation

Nelore non-lactating multiparous cows ranging between 5–7 years of age, with body condition scores ranging from 2.0 to 3.5, were subjected to FSH (n=10) or FSH/eCG (n=10) ovarian superstimulatory protocols, and a control group of synchronized cows was not subjected to any superstimulatory protocol (n=10). At a random stage of the estrous cycle, all cows received a progesterone-releasing vaginal insert (1.0 g, PRIMER**^®^**, Tecnopec, São Paulo, Brazil) and estradiol benzoate (2.5 mg, i.m., Estrogin^®^, Farmavet, São Paulo, Brazil) on day 0. The FSH protocol was commenced by administering pFSH (Folltropin-V**^®^**, Bioniche Animal Health, Belleville, ON, Canada) twice daily from day 5 to 8; a total of 200 mg was adminitered as follows: 40% on day 5, 30% on day 6, 20% on day 7, and 10% on day 8. All cows received 150 mg of d-cloprostenol (Prolise**^®^**, Tecnopec, São Paulo, SP, Brazil), i.m., twice on day 7 (7 am and 7 pm). The progesterone-releasing vaginal inserts were removed at 7 pm on day 8, and the cows were slaughtered at 7 am on day 9.

In the FSH/eCG approach, the last two doses of FSH were replaced with two doses of eCG (total dose of 400 IU, i.m., Novormon**^®^**, Syntex, Buenos Aires, Argentina). Additionally, blood samples were collected from their jugular vein, on day 8 at 7 pm and on day 9 at 7 am, to quantify the plasma concentration of LH and to ensure that no cow had undergone an endogenous LH surge (Figure 1).

### Follicular fluid and granulosa cells recovery

The preovulatory follicles were detected by ovarian ultrasonography 12 hours before slaughter. The ovaries were collected and stored in 0.9% saline solution at 4 °C, transported to the laboratory, and evaluated for the presence of *corpora lutea* or previous ovulation. The average diameter of each follicle, determined by measuring two perpendicular planes, was ascertained using a caliper. For synchronized cows, the dominant follicle was dissected (n= 10 follicles), whereas for cows subjected to the FSH protocol (n=10) and FSH/eCG protocol (n=10), the largest follicles were dissected. For all cows, the diameter of the follicles ranged between 11–14 mm.

After that, FF was aspirated, and any remaining cells were removed by centrifugation at 1000 ×*g* for 1 minute. The samples were stored at −80 °C. Further, the antral cavity was flushed repeatedly with cold saline, and GCs were recovered by centrifugation at 1200 × *g* for 1 minute. The pool of GCs from each follicle was placed in a buffer solution and homogenized using a Precellys-Tissue homogenizer (Bertin Corp.®) for three cycles of 30 seconds each. The recovered GCs were homogenized as previously described (Castilho et al., 2014) and total RNA was extracted with Trizol^®^ (Invitrogen, São Paulo, SP, Brazil) and stored at −80°C. Cross-contamination of theca and granulosa cells was tested by detection of mRNA encoding cytochromes P450 aromatase (CYP19) and 17b-hydroxylase (CYP17) in each sample by PCR, as previously described (Buratini et al., 2005). The mRNA relative abundance of CYP19A1 was assessed in GC to confirm the identity of the dominant and subordinate follicles, as described in Castilho et al. (2017) using the relative abundance of CYP19A1 combined with monitoring by ultrassom (Castilho et al., 2017).

### Intrafollicular concentration of triglycerides

To quantify the concentration of TG in FF, we used the kit from Laborlab® (São Paulo, Brazil), following the manufacturer’s instructions. TG was hydrolyzed by a specific lipase, producing glycerol and FA. Glycerol is oxidized to formaldehyde in the presence of acid. Ten microliters of the sample were used for incubation with 1ml of the standard reagents at 37 ºC for 5 minutes. After cooling, the color intensity was taken using a spectrophotometer. The experiments were performed in duplicate. Color intensity was determined by spectrophotometric analysis at 410 nm using ULTROSPEC 2000 (Pharmacia Biotech, Cambridge, England).

### RNA extraction and target gene expression

The mRNA expression of the 11 target genes was analyzed by reverse-transcription real-time PCR (RT-qPCR) (Table 1). According to the manufacturer’s protocol, the total RNA (1 µg/sample) from GCs was extracted with Trizol^®^ (Invitrogen, São Paulo, SP, Brazil), incubated with DNAse (1 UI/µg; Invitrogen, São Paulo, Brazil), and reverse transcribed using a High Capacity Kit (Applied Biosystems, São Paulo, Brazil) containing random primers.

**Table 1:** Relative mRNA abundance of genes related to lipid metabolism in GC from cows subjected to FSH or FSH/eCG superstimulatory protocols or synchronized group (n=10/group). The ^∆∆^Ct method with efficiency correction was used to calculate the relative expression values (target genes/*PPIA*) for each target gene, using one control sample as a calibrator.

**Gene**	**Forward sequence (5’-3’)**	**Reverse sequence (5’-3’)**	**Final concentration (mM)**	**Temp. annealing** **(°C)**
*ACSL1*	CCTCTTCTCCAGACCCAATTTC	GTTGTCACTGCAGAGTCTAAGG	300	60
*ACSL3*	ATAACTGGGATGGCGGAAAG	GACAGACAAGCTCAGCACTTA	300	60
*ACSL6*	CCTGTTGCCCAAGTCTATGT	CGTCCCTTCAATTCCTCTCTTC	300	60
*CPT1B*	CTGCTGAGAAGCACCAGAATA	GGTACTTGGAGACCACGTAAAG	300	60
*ELOVL5*	GAAGATCATCCGCGTCCTATG	CGTGATCTGGTGGTTGTTCT	300	62
*ELOVL6*	CTGTACTCCTGGTACTCCTACA	GCTCGCAAGGCATAGTAAGA	300	60
*FADS2*	GGGTGATGATGTGCTGGATT	CCCTGGACATCTGAAGAGAAAG	300	60
*FASN*	GTGGCCGACGTGGTAATAA	GGTGTGGATCCTTGAGATGTAG	300	60
*PPIA*	GCCATGGAGCGCTTTGG	CCACAGTCAGCAATGGTGATCT	300	60
*SCD*	GACCCTGGGCAAGTCATTTA	AAACTGCCCTTTGAGGTAGG	300	60
*SREBF1*	GACTACATCCGCTTCCTTCAG	CCAGGTCCTTCAGCGATTT	300	60

Relative qPCR analysis was performed using the QuantStudio™ 7 Flex. Reactions were carried out for 25 µL samples, with 1 µL of cDNA, 12.5 µL of Power Sybr Green PCR Master Mix (Applied Biosystems®), 1.25 µL of forward and reverse primer (300 mM), and 9 µL of water. The cycling conditions were 95°C for 10 minutes of initial denaturation, followed by 40 cycles of 95°C for 10 seconds and finally primer annealing and extension at 60°C for 1 minute. The reactions were optimized to achieve maximum amplification efficiency for each gene (90–110%). Each sample was analyzed in duplicate, and the specificity of each PCR product was determined by melting curve analysis. Positive controls (bovine fetal ovary extracts) and negative controls (water-replacing cDNA) were run on each plate.

To determine the most stable reference gene for detailed analyses of GCs, peptidylprolyl isomerase A (*PPIA*), glyceraldehyde-3-phosphate dehydrogenase (*GAPDH*), and histone H2AFZ (*H2AFZ*) amplification profiles were compared using the geNorm applet for Microsoft Excel (Ramakers et al., 2003), and the most stable gene was found to be *PPIA*. The ^∆∆^Ct method with efficiency correction was used to calculate the relative expression values (target genes/*PPIA*) for each target gene, using one control sample as a calibrator (Pfaffl, 2001). Primers used for the amplification of reference genes have been previously published (Machado et al., 2009).

### Statistical analysis

The effect of ovarian superstimulation on the mRNA abundance of the target genes and TG levels was transformed to the logarithmic scale, if it was not normally distributed, and tested by ANOVA. Mean comparisons were performed using the Tukey-Kramer HSD test. Non-transformed data is presented as mean ± SEM. Analyses were performed using the JMP software (SAS Institute, Cary, NC, USA). Differences were considered significant if *p* ≤0.05.

## Results

**Figure 2:**
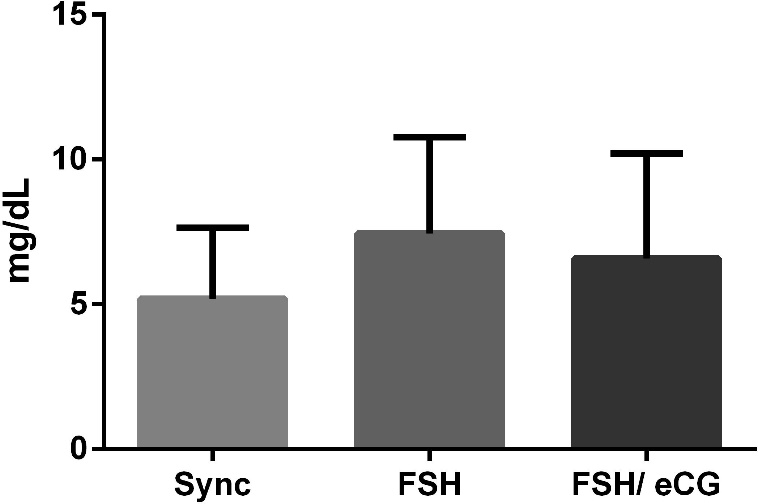
Effects of FSH and FSH/eCG on intrafollicular TG concentration from Nelore cows (n=10/group). Data is presented as means ± S.E.M, and differences were considered significant if P ≤0.05.

**Figure 3:**
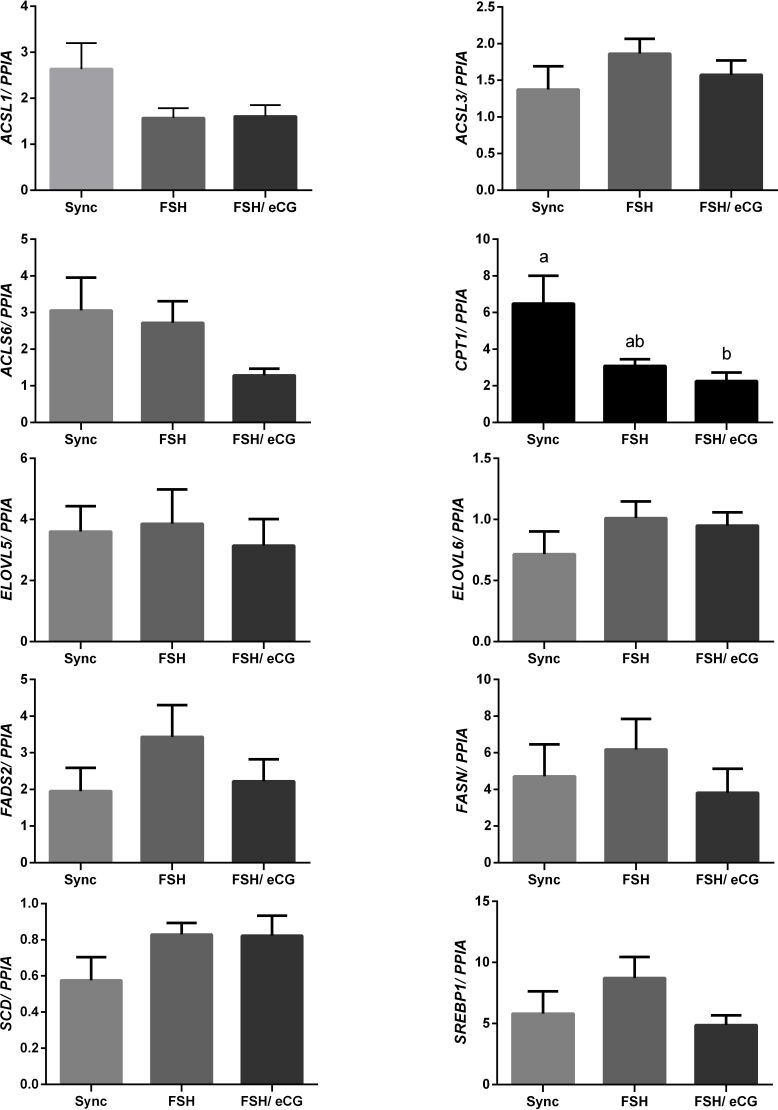
Relative mRNA abundance of genes related to lipid metabolism in GC from cows subjected to FSH or FSH/eCG superstimulatory protocols or synchronized group (n=10/group). The expression values are relative to a calibrator sample and calculated by the ^ΔΔ^Ct method with efficiency correction. Data is presented as means ± S.E.M, and different letters “a” and “b” are considered significantly different if P ≤0.05.

It was determined firstly that ovarian superstimualton did not affect TG levels in FF (P= 0.2948; Figure 2). Furthermore, only *CPT1B* mRNA abundance was affected by ovarian superstimulation (Figure 3), with a lower abundance for GCs from cows subjected to FSH/eCG (P= 0.0432) than the synchronized cows. The other target genes involved in lipid metabolism were not regulated by ovarian superstimulation (Figure 3).

## Discussion

During ovarian superstimulation with FSH alone or in combination with eCG, the follicular environment undergoes several changes (Castilho et al., 2014; Santos et al., 2017; Santos et al., 2018; Franchi et al., 2019); superstimulation was not found to modify follicular TG levels, nor affect mRNA abundance of genes related to lipid metabolism in GCs. Lipid metabolism is an essential mechanism to the reproductive process from the follicular to the embryo development (Ferguson and Leese, 2006; Prates et al., 2014; Gu et al., 2015; Bertevello et al., 2018).

FF is a product from plasma constituents and molecules secreted from GCs and theca cells, including molecular factors and act as a molecular exchange between the follicular cells, thereby influencing the development and quality of oocytes (Fortune et al., 2004; Guerreiro et al., 2018). TG is present in FF and is the main source of energy in ovarian somatic cells (Dunning et al., 2014). The TG levels in FF correlates to the size of the follicle; follicles >8 mm in diameter have decreased TG levels. The decreased levels could be explained by the mobilization of TG molecules of cumulus cells (CC) and oocytes (Annes et al., 2019). Oocytes store lipid droplets as an energy source (Aardema et al., 2011; Cran, 1985; Sturmey et al., 2009; Ferguson and Leese, 1999). Oocytes from follicles >8 mm in diameter exhibit lipid accumulation, which could be a step in preparation for proliferative activity in the growing embryo (Annes et al., 2019). We demonstrated that FSH or FSH/eCG protocols did not affect TG levels in FF in preovulatory follicles. We could not assess the oocyte lipid content profile, however (Franchi et al., 2019) demonstrated the increased expression of genes involved in FA synthesis and β-oxidation in preovulatory cumulus cells and oocytes from cows subjected to the FSH/eCG protocol.

FSH and FSH/eCG protocols increased the PC (34:2) (Santos et al., 2017) a phospholipid biomarker for embryo quality and cryopreservation success in cattle (Sudano et al., 2012). The combination of FSH/eCG demonstrated higher PC (34:8) and SM (16:0) than FSH alone, and phospholipids that seem to be structural units of functional membranes found in the FF microenvironment could reveal differences in membrane fluidity (Santos et al., 2017). Furthermore, similar cholesterol concentrations were found in FF from cows superstimulated with FSH/eCG and synchronized cows (Santos et al., 2018) suggesting that the ability of eCG molecules to bind to LHR influence the phospholipid profile but not alter cholesterol and TG concentrations.

Follicular development and eventual ovulation requires a great amount of energy, which has been reported to be sourced from the metabolism of lipid droplets in GCs (Ferguson and Leese, 2006; Aardema et al., 2011). Studies demonstrated the expression of enzymes relates to FA metabolism in GCs, that *in vitro,* FA metabolism sustains proper GCs, and one inhibitor of fatty acid synthesis (FAS) can reduce the production of progesterone, suggesting that ovarian steroidogenesis relies on lipid metabolism in GCs (Elis et al., 2015; Warzych et al., 2017).

Both FAS inhibitors and fatty acid oxidation (FAO) increased the expression of the gene encoding enzyme, carnitine palmitoyltransferase 1 B (*CPT1B). CPT1B* is a rate-limiting step in β-oxidation, as it is responsible for the influx of long-chain fatty acyl-CoAs in mitochondria cells (Dunning et al., 2014). The results of this study demonstrated lower *CPT1B* abundance in GCs from cows superstimulated with FSH/eCG, whereas *CPT1B* was found to be upregulated in cumulus cells and oocytes (Franchi et al., 2019). This contrast could be correlated to the function of cells. Once in the final maturation stage, the lipase activity increases and lipid droplet abundance decreases in bovine oocytes, suggesting the occurrence of β-oxidation (Dunning et al., 2014).

## Conclusion

The findings of this study demonstrate that FSH and FSH/eCG protocols maintained TG levels in FF and suggest no significant modulation of the transcriptional profile of genes involved in lipid metabolism. Consequently, the viability of GCs and a normal follicle microenvironment can be sustained.
